# Advanced Glycation End Products Induce Obesity and Hepatosteatosis in CD-1 Wild-Type Mice

**DOI:** 10.1155/2016/7867852

**Published:** 2016-01-31

**Authors:** Wael N. Sayej, Paul R. Knight III, Weidun Alan Guo, Barbara Mullan, Patricia J. Ohtake, Bruce A. Davidson, Abdur Khan, Robert D. Baker, Susan S. Baker

**Affiliations:** ^1^Digestive Diseases, Hepatology and Nutrition Center, Connecticut Children's Medical Center, Hartford, CT 06106, USA; ^2^University of Connecticut School of Medicine, Farmington, CT 06032, USA; ^3^Department of Anesthesiology, School of Medicine and Biomedical Sciences, State University of New York at Buffalo, Buffalo, NY 14214, USA; ^4^Veterans Administration Western New York Healthcare System, State University of New York at Buffalo, Buffalo, NY 14215, USA; ^5^Department of Microbiology and Immunology, School of Medicine and Biomedical Sciences, State University of New York at Buffalo, Buffalo, NY 14214, USA; ^6^Department of Surgery, University at Buffalo-SUNY School of Medicine, Buffalo, NY 14214, USA; ^7^Departments of Pathology and Anatomical Sciences, School of Medicine and Biomedical Sciences, Buffalo, NY 14228, USA; ^8^Department of Rehabilitation Science, University at Buffalo-SUNY School of Medicine, Buffalo, NY 14214, USA; ^9^Department of Pediatric Pathology, Women & Children's Hospital of Buffalo, Buffalo, NY 14203, USA; ^10^Digestive Diseases and Nutrition Center, Division of Pediatric Gastroenterology, Women & Children's Hospital of Buffalo, Buffalo, NY 14222, USA

## Abstract

AGEs are a heterogeneous group of molecules formed from the nonenzymatic reaction of reducing sugars with free amino groups of proteins, lipids, and/or nucleic acids. AGEs have been shown to play a role in various conditions including cardiovascular disease and diabetes. In this study, we hypothesized that AGEs play a role in the “multiple hit hypothesis” of nonalcoholic fatty liver disease (NAFLD) and contribute to the pathogenesis of hepatosteatosis. We measured the effects of various mouse chows containing high or low AGE in the presence of high or low fat content on mouse weight and epididymal fat pads. We also measured the effects of these chows on the inflammatory response by measuring cytokine levels and myeloperoxidase activity levels on liver supernatants. We observed significant differences in weight gain and epididymal fat pad weights in the high AGE-high fat (HAGE-HF) versus the other groups. Leptin, TNF-*α*, IL-6, and myeloperoxidase (MPO) levels were significantly higher in the HAGE-HF group. We conclude that a diet containing high AGEs in the presence of high fat induces weight gain and hepatosteatosis in CD-1 mice. This may represent a model to study the role of AGEs in the pathogenesis of hepatosteatosis and steatohepatitis.

## 1. Introduction

Nonalcoholic fatty liver disease (NAFLD) is defined histologically as a spectrum of liver diseases, ranging from hepatosteatosis to nonalcoholic steatohepatitis (NASH) and cirrhosis [1–3]. There is tremendous interindividual variability in the tendency to develop cirrhosis, the stage of NAFLD that is associated with the greatest liver-related morbidity and mortality [4]. The variable progression of NAFLD may be explained by the “multiple hit” hypothesis [3, 5, 6]. According to this hypothesis, a primary insult (“1st hit”), such as insulin resistance (IR), which may be primary or secondary to obesity [7–10], causes normal livers to accumulate fat [4, 11]. Fatty livers are unusually vulnerable to damage from various secondary insults and NASH develops when fatty livers experience a “2nd hit,” such as exposure to intestinal bacterial products and/or rapid mobilization of fatty acids from visceral (central) fat that induces inflammatory cytokines, which cause oxidative stress and further dysfunction [12, 13]. The liver is well known as a target organ and an important site for clearance and catabolism of macromolecules such as advanced glycation end products (AGEs) [14, 15]. AGEs have been shown to be elevated in patients with NASH [16–18].

AGEs are a heterogeneous group of molecules formed from the nonenzymatic reaction of reducing sugars with free amino groups of proteins, lipids, and/or nucleic acids. AGEs may be formed exogenously as a result of a chemical reaction by heating reducing sugars in the presence of fats or amino group containing compounds (Maillard reaction) and are commonly found in cooked Western Diets [19]. Additionally, tobacco smoke has been shown to be a source of toxic reactive AGEs [20] and tobacco-derived AGEs have been shown to increase risk for developing cancers and cardiovascular disease in former smokers [21]. AGEs are also produced endogenously during normal metabolism, aging [22–26] or due to hyperglycemia (e.g., diabetic patients) [27, 28]. Foods may contain up to 200 times the initial AGE content after cooking [29]. Processed foods have also been shown to be very high in AGEs and pose a significant problem in the Western world [19]. One of the most well-known AGEs, discovered in the 1960s, is hemoglobin A1c, which is also known as an Amadori product. Other well-known and investigated AGEs include* N* ^*ε*^-carboxymethyl-lysine (CML) [28, 30], pentosidine [31], methylglyoxal [32], and imidazolone [33–35]. CML has been shown to correlate with severity in patients with chronic liver disease (fibrosis and cirrhosis) [36].

The interaction of AGEs with their cell surface receptor (RAGE) has been shown to play a role in the pathogenesis of various devastating disorders [37] such as cancers [21, 38–42], Alzheimer's disease [43, 44], insulin resistance [45], diabetes and its complications including micro- and macrovascular disease, retinopathy [46], neuropathy, and nephropathy [47, 48], liver cirrhosis [49, 50], and nonalcoholic steatohepatitis (NASH) [16, 17, 51].

The detrimental effects of high fat content in the diet on obesity, diabetes, and cardiovascular disease are well known [52]. The detrimental effects of high levels of AGEs are also well studied in various diseases. The effects of AGEs on livers have only recently become a topic of discussion and research. Leung et al., utilizing an experimental mouse model, recently demonstrated that high dietary AGEs increase hepatic AGE content and exacerbate liver injury, inflammation, and liver fibrosis via oxidative stress and receptor for advanced glycation end product- (RAGE-) dependent profibrotic effects of AGEs on activated hepatic stellate cells [53]. We hypothesized that AGEs play an important role in the “multiple hit hypothesis” and thus the pathogenesis of hepatosteatosis and steatohepatitis. We also speculate that the presence of additional stressors (i.e., fat) is needed in the presence of high AGEs to cause significant hepatosteatosis and steatohepatitis.

Furthermore, there are a variety of inflammatory cytokines derived from adipose tissue, such as leptin, interleukin-6 (IL-6), and TNF-alpha (TNF-*α*). These cytokines have been found to be increased in obesity and may contribute to inflammation and hepatosteatosis. Myeloperoxidase (MPO) has been used to examine neutrophil infiltration and is regarded as a sign of tissue inflammation. The role that cytokines, AGEs, or their combination plays in the pathogenesis of NASH is unknown. Further, there is no data on the activity of MPO in NAFLD or how MPO might change with the development of disease. Based on this knowledge, we propose that AGEs play an important role in this “multiple hit” process and thus the pathogenesis of NAFLD, along with additional stressor such as fat and inflammatory cytokines.

## 2. Materials and Methods

### 2.1. The Animals

Four-week-old male CD-1 wild-type outbred mice purchased from Charles River Laboratories (Wilmington, MA) were used for this study. The mice were housed at an animal facility at the Buffalo Veterans Affairs Medical Center. All procedures were approved by the Institutional Animal Care and Use Committee at the Buffalo Veterans Affairs Hospital and complied with all State, Federal, and National Institutes of Health regulations.

### 2.2. The Diets

**Figure 1:**
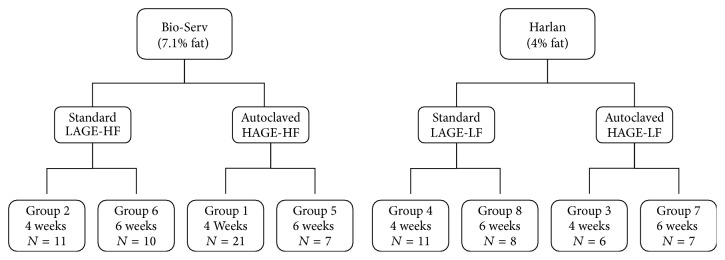
Flowchart of mice groups based on chow assigned. *N* shows the number of mice in each group. HAGE-HF: high AGE-high fat, HAGE-LF: high AGE-low fat, LAGE-HF: low AGE-high fat, and LAGE-LF: low AGE-low fat.

The mice were fed a standard rodent diet, Purina 5L79 (LabDiet®, St. Louis, MO) for the 1st 4 weeks of life. Once in our animal facilities (at 4 weeks of age), the mice were fed* ad libitum* two different rodent chows from Bio-Serv® (Frenchtown, NJ, USA) and Harlan® Laboratories (Madison, WI, USA) until sacrifice. The first diet from Bio-Serv, a semipurified standard diet, AIN-93G rodent diet (Stk# F3542), was processed with 100°C heating for 20–60 seconds. To create the high AGE diet, the chow was heated for additional 30 minutes at 125°C. The second diet from Harlan Laboratories, also a standard rodent diet (Stk# Tek 70001.15), was processed with heating at 60–80°C for 20–30 seconds and additional 5 seconds at 65–85°C. To create the high AGE diet, the chow was heated for additional 30 minutes at 125°C.  Table 1 summarizes the macro- and micronutrients of each of the diets as well as the AGE levels in each of the diets.  Figure 1 is a flow diagram of the mouse groups based on diet (chow) given.

The groups and diets were designated the following names: groups 1 and 5 received Bio-Serv's high AGE/high fat (HAGE-HF), groups 2 and 6 received Bio-Serv's low AGE/high fat (LAGE-HF), groups 3 and 7 received Harlan's high AGE/low fat (HAGE-LF), and groups 4 and 8 received Harlan's low AGE/low fat (LAGE-LF) chow. We were not sure if 4 weeks was enough time for the diets to change the phenotype and therefore, we decided to give the diets for 4 weeks and 6 weeks to determine if that made a difference in the phenotypes. Additionally, we wanted to see if there was progression of the inflammation from 4 to 6 weeks. Groups 1–4 received the diets for a total of 4 weeks and sacrifice was at 8 weeks ± 2 days of age. Groups 5–8 received the diets for a total of 6 weeks and sacrifice was at 10 weeks ± 2 days of age. The mice were fed* ad libitum*. The mice were weighed at time of arrival to our facilities, weekly, and at time of sacrifice to measure weight gain during the period of the study.

### 2.3. Diet AGE Levels

Samples of the diets were crushed and sent to Dr. Peter Stahl at MicroCoat Biotechnologie GmbH Â, Bernried, Germany. The chows that were exposed to higher temperatures and for a longer period of time (autoclaved for 30 minutes at 125°C) contained 4-5-fold higher AGE levels than the chow as prepared by the manufacturer. AGE levels and the nutrient content in each of the diets are listed in Table 1.

### 2.4. Mouse Harvest and Organ Preparations

Mice were weighed prior to sacrifice. The mice were anesthetized with 2% halothane in oxygen at a rate of 5 L/min. After induction of anesthesia, a midline abdominal/thoracic incision was made to allow full exposure of the abdominal viscera. The epididymal fat pads were resected and weighed. The inferior vena cava was transected and the heart was flushed with 5 mL Hank's Balanced Salt Solution (1xHBSS) with Mg^+^ and Ca^+^ (Life Technologies, Grand Island, NY) to completely exsanguinate the mice.

The livers were harvested and weighed. A sample from each liver was immediately immersed in formaldehyde. The remaining liver was washed with cold HBSS and suspended in cytokine homogenate buffer (150 mM NaCl, 15 mM Tris, 1 mM CaCl_2_·2H_2_O, and 1 mM MgCl_2_·6H_2_O, adjusted to pH 7.4) plus 100x protease inhibitor cocktail 1 (Calbiochem, La Jolla, California) to a total volume of 10 mL and homogenized on ice using a Polytron® Homogenizer (Kinematica Inc., Bohemia, NY). The samples were centrifuged at 40,000 g for 15 minutes at 4°C (Avanti® J-E Centrifuge and JA-20 rotor, Beckman Coulter, Fullerton, CA). The supernatant between the pellet and the superficial layer (consisting mainly of fat and other tissues) was collected, transferred in aliquots to 1.5 mL tubes, and stored at −80°C for cytokine analysis by ELISA at a later time.

### 2.5. Liver Histology

The tissue in formaldehyde was embedded in paraffin blocks, sliced into 4 *μ*m sections with a microtome, and fixed on glass slides. After they were deparaffinized with xylene and ethanol, they were stained with hematoxylin and eosin (H&E) and trichrome stains. An experienced pathologist performed histologic analysis in blinded fashion. The slides were examined and photographed under a light microscope (Olympus BH-2 microscope, Olympus America Inc., Center Valley, PA).

### 2.6. Myeloperoxidase Assay

The pellets that remained after homogenization were suspended in myeloperoxidase homogenate buffer (0.5% hexadecyltrimethylammonium bromide (HTAB) (13.7 mM), 5 mM EDTA, and 50 mM potassium phosphate (KH_2_PO_4_) buffer, adjusted to pH = 6), sonicated for 1 minute at 50% using the Branson Sonifier 450 with a microtip probe (Branson Ultrasonics Corporation, Danbury, CT), covered with aluminum foil, incubated in a 55°C water bath for 2 hours, and then centrifuged again at 40,000 g for 15 minutes at 4°C. The supernatants were reserved, with total volume measured, and stored at −80°C until the MPO activity assay was performed.

MPO activity was measured by continuously monitoring the H_2_O_2_-dependent oxidation of o-dianisidine dihydrochloride (ODH) using a SpectraMax 190 and Software Max (Molecular Devices Corporation, Sunnyvale, CA) at 460 nm, time 90 seconds, and intervals of 2 seconds which gave us 46 reads. MPO activity was expressed as the absorbance change at 460 nm (ABS) per minute over the linear portion of the curve and normalized to the total volume extracted from the liver.

### 2.7. Cytokine Analysis

Quantification of leptin, interleukin-6 (IL-6), and TNF-alpha (TNF-*α*) in cell free supernatants was determined by enzyme-linked immunoassays (ELISA) with ELISA kits from R&D systems (Minneapolis, MN).

### 2.8. Statistical Analysis

All data are expressed as mean ± SEM. We used Kruskal-Wallis tests followed by Dunn's multiple comparisons test and Mann-Whitney test (nonparametric) to determine statistical significance when comparing 2 groups, with *α* error set to 0.05, to control for multiple comparisons using GraphPad Prism 5.0® software (GraphPad Software, Inc., San Diego, CA). Values for myeloperoxidase and cytokines were log transformed prior to performing statistical analysis to normalize data. Pearson correlation was performed to determine the linear correlation between MPO activity and weight gain percentage (%) to initial weight or fat pad % of final weights. A *P* value of <0.05 was considered statistically significant.

## 3. Results

### 3.1. Effect of Diet on Weight Gain

**Figure 2:**
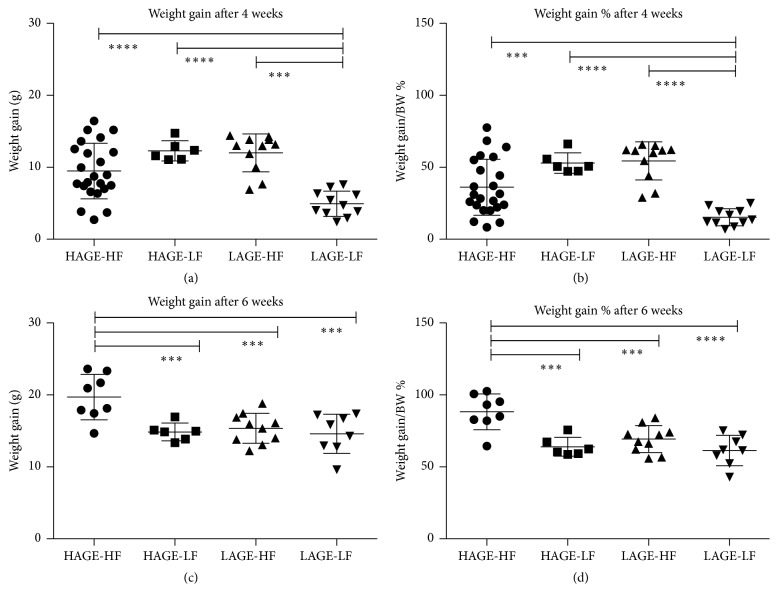
Weight gain comparisons. (a) Weight gain at 4 weeks in each group based on the different diets. (b) Weight gain as percentage of final body weight at 4 weeks. (c) Weight gain at 6 weeks in each group based on the different diets. (d) Weight gain as percentage of final body weight at 6 weeks. ^*∗∗∗∗*^ *P* < 0.0001, ^*∗∗∗*^ *P* < 0.001.

To determine the effects of each diet on weight gain, the mice were weighed at arrival (4 weeks old), weekly, and at time of sacrifice (8 weeks and 10 weeks old). To take into consideration variations in initial weight of mice, the weight gain (grams) was calculated as a percentage of the final weight of the mice. After 4 weeks on the diets, LAGE-LF (group 4) mice had significantly less weight gain than the other 3 groups. After 6 weeks on the diets mice that were fed the HAGE-HF chow (group 5) had the most weight gain (20.4 g ± 1.0; 88.3% ± 4.4) compared to the other three groups, *P* < 0.0001. Additionally, there was a significant difference in weight gain when HAGE-HF (group 5) was compared to the individual groups: versus LAGE-HF (group 6) (15.4 g ± 0.7; 69.3% ± 4), *P* = 0.001; versus HAGE-LF (group 7) (14.8 g ± 0.5; 64% ± 2.7), *P* = 0.0005; and versus LAGE-LF (group 8) (14.6 g ± 1.0; 61.4% ± 3.7), *P* = 0.0002.  Table 2 summarizes the weight gain means ± standard error (SEM) for each group of mice according to the diets that they were given and Figure 2 demonstrates the differences in weight gain and weight gain as % of final weight at 4 weeks and 6 weeks.

### 3.2. Epididymal Fat Pads

**Figure 3:**
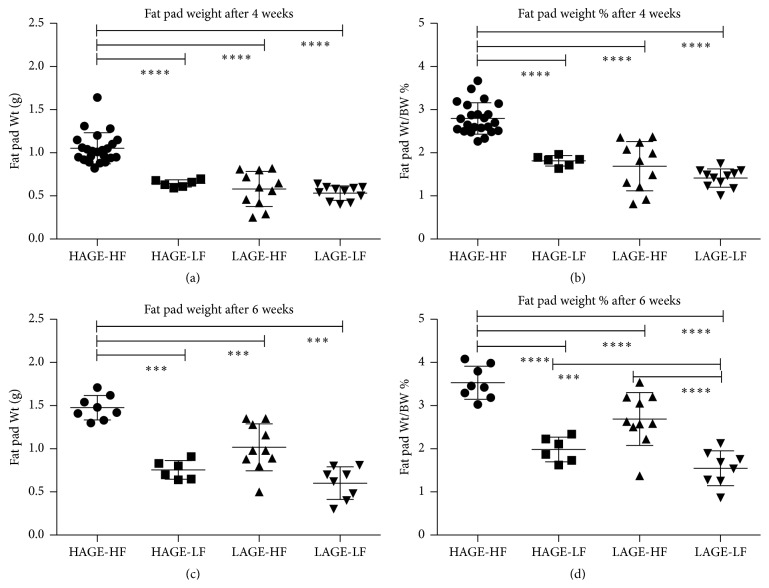
Epididymal fat pad weight comparisons. (a) Epididymal fat pad weight at 4 weeks of comparison. (b) Epididymal fat pad weight as percentage of final weight at 4 weeks. (c) Epididymal fat pad weight at 6 weeks of comparison. (d) Epididymal fat pad weight as percentage of final weight at 6 weeks. ^*∗∗∗∗*^ *P* < 0.0001, ^*∗∗∗*^ *P* < 0.001.

Adipose tissue stores lipids in the form of triglycerides when energy intake exceeds expenditure. Epididymal fat pads from mice fed the HAGE-HF diet for 4 and 6 weeks (groups 1 and 5) were heavier and a higher percent of body weight compared to those fed LAGE-HF, HAGE-LF, or LAGE-LF. There was no difference between the LAGE-HF group and the HAGE-LF group in fat pad weight or percent body weight at 4 and 6 weeks. Fat pads from the LAGE-LF group were small in both average weight and percent body weight at 4 and 6 weeks when compared to either the LAGE-HF group or the HAGE-LF group.  Table 2 summarizes the epididymal fat pad weights expressed in mean ± SEM and as mean percentage of final body weights ± SEM for each group of mice according to the diets that they were given and Figure 3 demonstrates the differences in epididymal fat pad weight and weight as percent of final weight at 4 weeks and 6 weeks.

### 3.3. Effects of Diet on Liver Weight

Table 2 shows that whole wet liver weights were not significantly different in any of the groups after 4 and 6 weeks on the diets.

### 3.4. Histology

**Figure 4:**
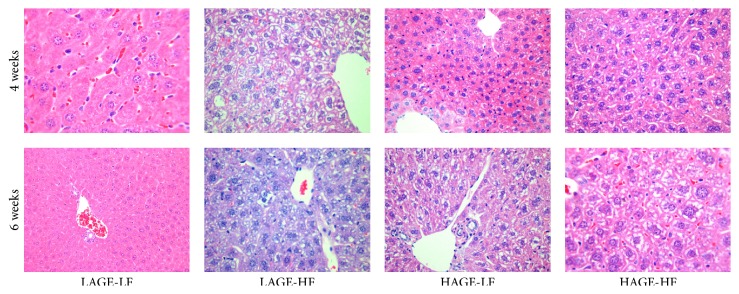
Hematoxylin and eosin staining of liver tissues showing normal histology in the LAGE-LF groups after 4 and 6 weeks; varying degrees of steatosis in all other groups and mixed micro- and macrovesicular steatosis and mild steatohepatitis in the HAGE-HF groups after 4 and 6 weeks.

Histology of liver shows that microvesicular steatosis had developed in all groups except for those fed the LAGE-LF. After 6 weeks on the chow, the mice in the HAGE-HF also had evidence of progression to macrovesicular steatosis. Steatohepatitis (inflammation) was seen in approximately 40% of the mice that received the HAGE-HF chow after 6 weeks. No inflammation was seen in the LAGE-LF groups.  Figure 4 shows H&E staining representing a randomly selected set of slides per group of mice after 4 weeks and 6 weeks on the diets, respectively. Trichrome staining did not show evidence of fibrosis in any of the groups.

### 3.5. Myeloperoxidase Activity

**Figure 5:**
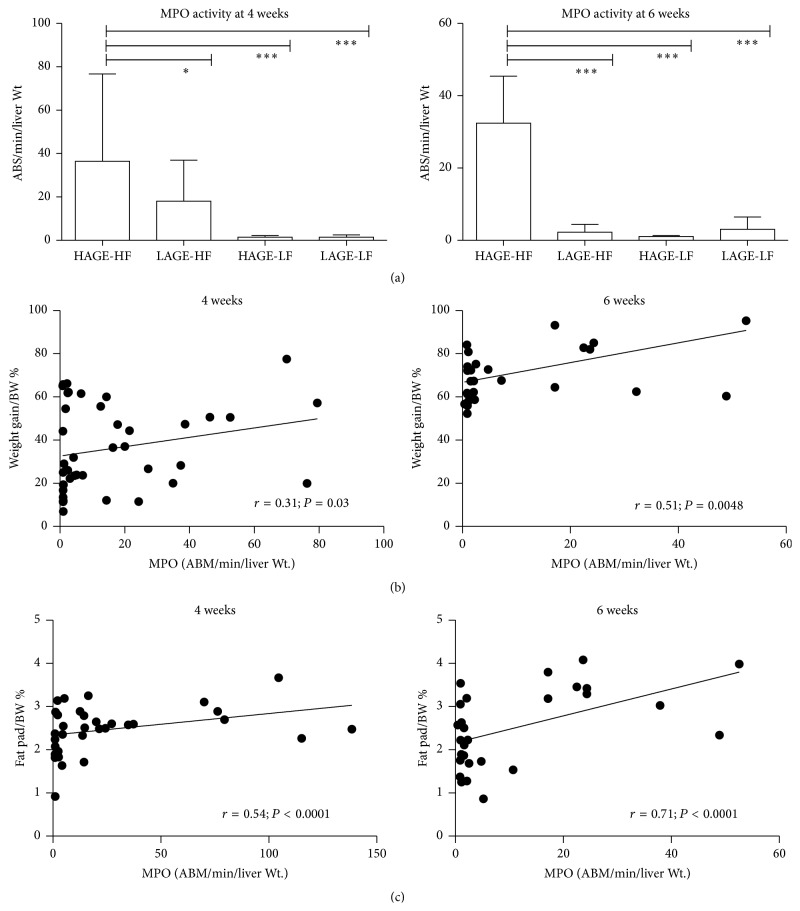
Myeloperoxidase activity in liver tissues. (a) MPO activity is highest in the HAGE-HF groups after 4 and 6 weeks. (b) MPO activity strongly correlates with weight gain. (c) MPO activity strongly correlates with epididymal fat pad weights.

Liver MPO activity was significantly higher in the groups that received the HAGE-HF diet than the other groups after 4 weeks and 6 weeks; *P* < 0.0001 and *P* = 0.0001, respectively. This is consistent with the acute liver inflammation observed with histology. There was no significant difference in the other groups after 4 weeks or 6 weeks.  Figure 5 summarizes the MPO activity results expressed as (ABS/min/liver) ± SEM. MPO activity also significantly correlated with weight gain and epididymal fat pad weight at 4 and 6 weeks.

### 3.6. Cytokine Analysis

**Figure 6:**
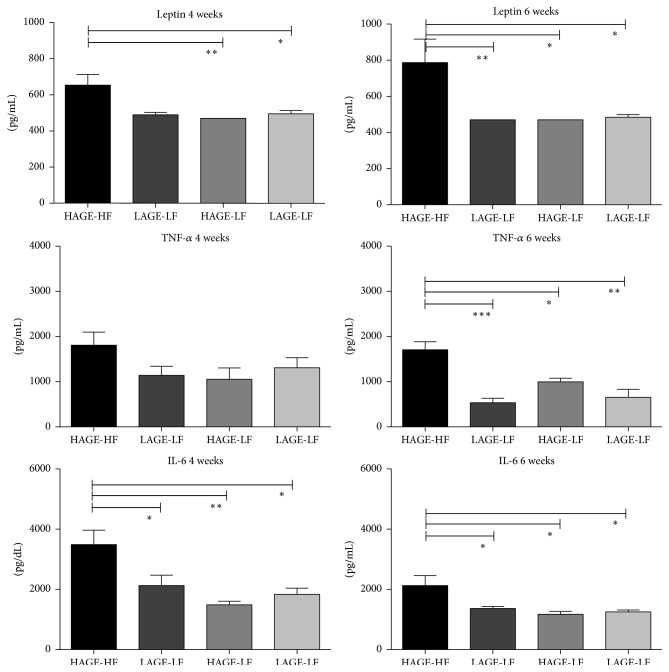
Leptin and cytokine levels (IL-6 and TNF-*α*) after 4 and 6 weeks on the diets. Consistent with the weight gain and epididymal fat pad weights, leptin levels were significantly higher after 4 and 6 weeks in the HAGE-HF group compared to the other groups. Also consistent with the liver histology and presence of inflammation after 6 weeks on the diet, IL-6 and TNF-*α* levels were significantly higher in the HAGE-HF group after 6 weeks on the diet.

The proinflammatory cytokine tumor necrosis factor-*α* (TNF-*α*) is synthesized and released by adipocytes [54] and may play a role in the induction of insulin resistance. [55, 56] TNF-*α* levels were not significantly different after 4 weeks on the diets (*P* = 0.47) (Figure 6). However, after 6 weeks, the HAGE-HF chow fed mice had higher TNF-*α* levels compared to the LAGE-HF group (*P* = 0.0002), the HAGE-LF group (*P* = 0.01), and the LAGE-LF group (*P* = 0.006), respectively (Figure 6). Additionally, the HAGE-LF group had higher levels than the LAGE-HF and LAGE-LF groups, which may indicate that the AGE content and not the fat content is what contributed to the elevated levels of TNF-*α* (Figure 6).

TNF-*α* induces the release of IL-6 from several cell types. Circulating IL-6 stimulates the hypothalamic-pituitary-adrenal (HPA) axis, activation of which is associated with central obesity, hypertension, and insulin resistance. As expected, IL-6 levels were significantly elevated in the HAGE-HF groups compared to the other groups at 4 weeks (*P* = 0.02) and at 6 weeks (*P* = 0.005), respectively (Figure 6).

## 4. Discussion

Mice fed the HAGE-HF diet accumulated more fat than the other group as shown by the larger weight gain and larger epididymal fat pad. There was no significant difference in liver weight among all groups. Some HAGE-HF fed mice showed evidence of hepatosteatosis and steatohepatitis. There was mild, if any, hepatosteatosis seen in the LAGE-HF fed mice. Diets high in fat content are associated with weight gain and obesity and energy consumed in excess of requirements is stored in the form of triglycerides in adipose tissues. It was not surprising that the mice fed the HAGE-HF developed hepatosteatosis and/or steatohepatitis and the mice that were fed the LAGE-LF diet had normal liver histology. However, the mice that were fed the HAGE-LF had similar outcomes as the mice that were fed the LAGE-HF in terms of weight gain and epididymal fat pad findings. There was evidence of mild hepatosteatosis in these groups. Additionally, mice that were fed the HAGE-HF had significantly more weight gain and higher epididymal fat pad weights compared to the HAGE-LF and the LAGE-HF fed mice. This suggests that AGEs likely played a role in the development of hepatosteatosis.

In addition to the liver fat, MPO activity was increased in mice on HAGE-HF. As MPO is an indicator of organ inflammation with acute neutrophilic infiltration, this indicates that AGEs may contribute to liver inflammation.

Leptin is an adipose derived protein hormone that plays important roles in regulating food intake and energy expenditure, including appetite and metabolism [57]. It is a circulatory biomarker that is proportional to body fat. Leptin levels control food intake and energy expenditure by acting on receptors in the mediobasal hypothalamus [58]. In obesity, a putative resistance to leptin develops, which prevents the normal negative feedback control mechanism responsible for adipocyte leptin production from functioning properly [59]. This leads to elevated plasma levels of leptin. The increased leptin levels found in the HAGE-HF fed mice suggest the leptin resistance to obesity and fatty liver, as well as the inflammation in these fatty livers. It is possible, however, that other adipokines may play a role.

Finally there is clear evidence of a heightened proinflammatory state in the mice that were fed the HAGE-HF chow. In obesity, inflammation occurs due to altered metabolic homeostasis in accordance with dietary intake. This leads to upregulation of proinflammatory genes (TNF-*α*, IL-6, CRP, leptin, and IL-1*β*) and downregulation of anti-inflammatory genes (IL-10, IL-Ra, and adiponectin) encoding for the respective cytokines, chemokines, and adipokines [60]. The proinflammatory cytokine tumor necrosis factor-*α* (TNF-*α*) is synthesized and released by adipocytes [54] and has been shown to play a role in the induction of insulin resistance [55, 56]. Additionally, TNF-*α* and IL-6 are strong mediators of insulin resistance, which is the best-examined link in the pathogenesis of polycystic ovarian syndrome [61]. Lipopolysaccharide (LPS) has been shown to be one of the major players in nonalcoholic fatty liver disease pathogenesis and progression. It has been shown to induce a proinflammatory and profibrogenic phenotype of NASH via LPS induced TNF-*α*-dependent transcription of TNF-*α*, IL-6, and IL-1*β* [62]. TNF-*α* and IL-6 are both elevated in the liver tissues of mice fed a HAGE-HF chow. This is consistent with MPO activity and histology of the liver, indicative of acute neutrophilic infiltration in the liver. Elevated levels of TNF-*α* and the soluble receptors of this cytokine are consistent with a continuing inflammatory state. TNF-*α* enhances the release of IL-6 from in a number of cell types especially adipocytes. IL-6 is a potent inducer of the hepatic acute phase response observed in many systemic inflammatory responses (i.e., sepsis). It has also been proposed that IL-6 released from adipocytes stimulates hepatic synthesis of C-reactive protein (CRP) in obesity [63–65]. Additionally, increased circulating IL-6 levels stimulate the hypothalamic-pituitary-adrenal (HPA) axis, which plays an important role in control of central obesity, hypertension, insulin resistance, and PCOS. Hypothalamic inflammation has been shown to be associated with high fat diet intake in rats and increased expression of hypothalamic TLR-4, NF-*κβ*, TNF-*α*, IL-1*β*, and IL-6 [66].

Our study has some limitations. We only investigated the local inflammation in the livers but did not evaluate the systemic inflammatory markers. Thus, we could not define the relationship between systemic inflammation and NAFLD.

## 5. Conclusions

Diets containing high AGEs and fat contribute to obesity and NAFLD in CD-1 mice, which are associated with increased inflammation of the liver. This suggests that dietary AGEs coupled with high fat play an important role in the development of obesity and NAFLD, possibly by promoting a proinflammatory state. These findings warrant further study as they may represent a model that can be used to study the pathogenesis of NAFLD.

## Figures and Tables

**Table 1:** Dietary macro- and micronutrients and AGE levels.

	Bio-Serv		Harlan Laboratories	
	Autoclaved	Not autoclaved	Autoclaved	Not autoclaved
Mouse groups	*Groups 1* &* 5*	*Groups 2* &* 6*	*Groups 3* & *7*	*Groups 4* & *8*
Diet designation	HAGE-HF	LAGE-HF	HAGE-LF	LAGE-LF
AGE^*∗*^ (ng/g of chow)	119.949	22.969	110.49	34.105

Nutrients (%)				
Protein	18.4	18.4	24	24
Fat	7.2	7.2	4	4
Carbohydrate	58.6	58.6	46.16	46.16
Other (fiber)	15.8	15.8	5	5
Total calories	3.9	3.9	3.82	3.82

Micronutrients (per kg of chow)				
Biotin	0.2 mg/Kg	0.2 mg/Kg	0.38 mg/Kg	0.38 mg/Kg
Folate	2.0 mg/Kg	2.0 mg/Kg	3.21 mg/Kg	3.21 mg/Kg
Niacin	30.0 mg/Kg	30.0 mg/Kg	60.42 mg/Kg	60.42 mg/Kg
Pyridoxine	6.0 mg/Kg	6.0 mg/Kg	14.13 mg/Kg	14.13 mg/Kg
Riboflavin	6.0 mg/Kg	6.0 mg/Kg	7.89 mg/Kg	7.89 mg/Kg
Thiamin	5.0 mg/Kg	5.0 mg/Kg	28.08 mg/Kg	28.08 mg/Kg
Vitamin A	4000 IU	4000 IU	15.79 IH/g	15.79 IH/g
Vitamin B12	25.0 mcg	25.0 mcg	51.2 mcg/Kg	51.2 mcg/Kg
Vitamin E	75 IU	75 IU	94.72 IU/Kg	94.72 IU/Kg
Vitamin D3	1000 IU	1000 IU	2.98 IU/g	2.98 IU/g

^*∗*^AGE: measured using CML- (*N* ^6^-*carboxymethyl-lysine-*) *s*ensitive ELISA.

**Table 2:** Effect of the diets on weight gain, epididymal fat pad, and liver weights.

	HAGE-HF	LAGE-HF	HAGE-LF	LAGE-LF	*P* value^a^

	Group 1	Group 2	Group 3	Group 4	
	*n* = 20	*n* = 11	*n* = 6	*n* = 11	

Four weeks					
Weight gain (g)	10.4 ± 0.7	12 ± 0.8	12.3 ± 0.6	4.9 ± 0.5	<0.0001
Weight % gain^*∗*^	40 ± 4	54.4 ± 4	53 ± 3	15.3 ± 3	<0.0001
Fat pad weight (g)	1.1 ± 0.04	0.6 ± 0.06	0.7 ± 0.02	0.5 ± 0.03	<0.0001
Fat pad weight %^*∗∗*^	2.8 ± 0.4	1.7 ± 0.6	1.8 ± 0.1	1.3 ± 0.3	<0.0001
Liver weight (g)	2.1 ± 0.06	1.8 ± 0.1	2 ± 0.04	2.2 ± 0.04	NS
Liver weight %^*∗∗*^	5.5 ± 0.1	5.4 ± 0.2	5.6 ± 0.1	5.8 ± 0.1	NS

	Group 5	Group 6	Group 7	Group 8	
	*n* = 7	*n* = 10	*n* = 6	*n* = 8	

Six weeks					
Weight gain (g)	20.4 ± 1.0	15.4 ± 0.7	14.8 ± 0.5	14.6 ± 1.0	<0.0001
Weight % gain^*∗*^	88.3 ± 4.4	69.3 ± 3	64 ± 2.7	61.4 ± 3.7	<0.0001
Fat pad weight (g)	1.6 ± 0.05	0.9 ± 0.05	0.8 ± 0.03	0.5 ± 0.04	<0.0001
Fat pad weight %^*∗∗*^	3.7 ± 0.3	2.3 ± 0.4	2.1 ± 0.2	1.3 ± 0.3	<0.0001
Liver weight (g)	2.1 ± 0.06	2 ± 0.01	2 ± 0.04	2 ± 0.02	NS
Liver weight %^*∗∗*^	5.2 ± 0.2	5.3 ± 0.1	5.4 ± 0.1	5.2 ± 0.1	NS

Values are presented with mean ± SEM.

^a^We used nonparametric Kruskal-Wallis tests followed by Dunn's multiple comparisons test. Results were considered statistically significant at *P* < 0.05.

^*∗*^Weight % gain is expressed as weight gain in relation to initial weight of animal (final weight-initial weight/initial weight).

^*∗∗*^Fat pad weight % and liver weight % are expressed as weight of fat pad or liver in relation to final weight of animal (fat pad or liver weight/final weight).

## Abbreviations

### Abbreviations

BS: Bio-Serv
HT: Harlan-Tek
AGEs: Advanced glycation end products
HAGE: High AGE
LAGE: Low AGE
HF: High fat
LF: Low fat
IL-6: Interleukin-6
TNF-*α*: Tumor necrosis factor-*α*
MPO: Myeloperoxidase
RAGE: Receptor for AGE
ELISA: Enzyme-linked immunoassay
NAFLD: Nonalcoholic fatty liver disease
SEM: Standard error of the mean.
